# Pathogenesis of diabetic cardiomyopathy and emerging therapeutic strategies: a network-based perspective

**DOI:** 10.3389/fcdhc.2026.1823872

**Published:** 2026-05-18

**Authors:** Haoqing Ren, Hengli Lai

**Affiliations:** 1 Jiangxi Medical College, Nanchang University, Nanchang, China; 2 Department of Cardiology, Jiangxi Provincial People’s Hospital, The First Affiliated Hospital of Nanchang Medical College, Nanchang, China

**Keywords:** diabetic cardiomyopathy, ferroptosis, metabolic memory, metabolic-structural coupling, networked injury, pathogenesis, senolytics, SGLT2 inhibitors

## Abstract

Diabetic cardiomyopathy (DCM) is a distinct myocardial disease in diabetic patients, independent of coronary artery disease or hypertension, and a leading cause of heart failure. Its pathogenesis has evolved from a linear “glucolipotoxicity” model to a complex, dynamic network involving metabolic disturbances, mitochondrial dysfunction, oxidative stress, chronic inflammation, diverse programmed cell death pathways, cellular senescence, and cardiac autonomic neuropathy. These interconnected events are amplified through positive feedback loops, driving progressive myocardial damage. This review provides a systems-level synthesis of the networked pathogenesis of DCM, integrating evidence across core mechanistic components and their dynamic interactions. We discuss how metabolic disturbances initiate and propagate injury signals through mitochondrial dysfunction, inflammatory activation, and alterations in cardiomyocyte fate—including apoptosis, necroptosis, ferroptosis, and senescence. Distinct from prior reviews, this article explores two emerging integrative concepts: “metabolic–structural coupling”—abnormal physical interactions between metabolic molecules and myocardial structural proteins—and “metabolic memory”—epigenetically mediated persistence of injury despite glycemic control. We further provide a horizontal comparison and stage-specific assessment of emerging therapeutic strategies, including the pleiotropic network effects of SGLT2 inhibitors, the cardiovascular benefits of GLP-1 receptor agonists, targeting ferroptosis (e.g. irisin, alpha-lipoic acid), senolytics (e.g. dasatinib + quercetin), and the modulation of lipid droplet dynamics and the farnesoid X receptor (FXR). Finally, we critically evaluate the current evidence levels and translational barriers for these emerging therapies—from preclinical targets to completed randomized controlled trials—and propose a forward-looking roadmap to advance precision medicine in DCM. This roadmap emphasizes the multi-omics dissection of clinical heterogeneity, the development of mechanism-based biomarkers, and the validation of targeted combination therapies in well-designed clinical trials, with metabolic-structural coupling and metabolic memory serving as integrative links between classical pathways and disease persistence.

## 1 Introduction

Cardiovascular complications of diabetes have become a major global public health challenge. Beyond accelerating atherosclerosis, diabetes can directly damage the myocardium, leading to a specific cardiomyopathy—diabetic cardiomyopathy (DCM) (1). DCM is characterized by progressive structural and functional cardiac abnormalities in the absence of significant coronary artery disease, hypertension, or valvular heart disease. Its clinical course typically begins with subclinical diastolic dysfunction and may eventually progress to symptomatic heart failure, regardless of ejection fraction (2, 3), “Pure” DCM (excluding hypertension and CAD) occurs in 3%-17% of diabetic patients, depending on diagnostic criteria. However, up to 64% have some cardiac abnormality when comorbidities are included.

For decades, the “glucolipotoxicity” model dominated the pathophysiological understanding of DCM, emphasizing direct myocardial injury caused by high glucose and free fatty acids (4, 5). Although instructive, this relatively linear framework fails to fully explain the complexity of DCM phenotypes, particularly the phenomenon where cardiac injury continues to progress even after strict glycemic control in advanced stages (6). This limitation has prompted a profound shift in perspective. Recently, research has moved from isolated “toxic pathways” toward understanding a highly interconnected, dynamically interacting molecular network. This network is initiated by metabolic disturbances and subsequently triggers mitochondrial dysfunction, redox imbalance, chronic low-grade inflammation, multiple forms of programmed cell death, epigenetic reprogramming, and abnormal intercellular communication—events that are not linearly transmitted but intertwined, forming complex feedback loops (3, 7–9).

Compared with previous reviews, the novel contributions of this article are: (1) it systematically integrates the emerging concepts of “metabolic–structural coupling” and “metabolic memory” into a unified network framework, elucidating their interactions with classical pathways; (2) it provides a horizontal comparison and stage-specific assessment of emerging therapeutic targets, clarifying their evidence levels and developmental stages; (3) it reinterprets the mechanisms of pleiotropic drugs such as SGLT2 inhibitors from a network biology perspective. This review aims to systematically construct a network-based framework of DCM pathogenesis and, on this basis, provide a comprehensive overview of emerging therapeutic strategies.

## 2 Core network components of DCM pathogenesis

### 2.1 Metabolic disturbances: the initiator and driver of the network

Below briefly synthesize these well-established pathways; detailed mechanistic descriptions are available in recent comprehensive reviews. The heart possesses remarkable metabolic flexibility, efficiently switching between fatty acid oxidation and glucose oxidation to meet energy demands (10, 11), Diabetes disrupts this balance, inducing “pathological metabolic remodeling”, which serves as the initial driver of the entire injury network.

In terms of glucose metabolism, myocardial insulin resistance leads to reduced membrane translocation of glucose transporter 4 (GLUT4) and impaired glucose uptake (3, 12, 13) GLUT4 is the main insulin-responsive glucose transporter in cardiac cells. The knockout of the cardiac-specific GLUT4 gene leads to the loss of insulin-induced glucose uptake function, confirming its crucial role in the pathogenesis of dilated cardiomyopathy. Persistent hyperglycemia mediates damage through four parallel pathways, AGE-RAGE signaling (activating NF-κB-driven inflammation), PKC activation (impairing vascular and metabolic regulation), polyol pathway flux (depleting antioxidant capacity), and hexosamine pathway flux (causing O-GlcNAc modification of key proteins) (14–19).

Lipid metabolism abnormalities and lipotoxicity also play critical roles. The heart exhibits increased uptake and β-oxidation of free fatty acids (FFAs), while glucose oxidation is suppressed. When FFA supply exceeds mitochondrial oxidative capacity, toxic lipid intermediates such as ceramides and diacylglycerols (DAG) accumulate. Ceramides are potent inducers of apoptosis, promoting insulin resistance, oxidative stress, and mitochondrial dysfunction (20). DAG can persistently activate PKC, further impairing insulin signaling and forming a vicious cycle (21, 22). Together, these abnormalities lead to a “metabolic rigidity” that reduces ATP production efficiency and causes energy deficiency, forming the energetic basis for early diastolic dysfunction in DCM. The important point is that these metabolic disorders do not completely disappear even after blood sugar levels return to normal. That is, although blood sugar is under control, through “metabolic memory”, epigenetic modifications will still continue to cause functional disorders.

### 2.2 Mitochondrial dysfunction and oxidative stress: energy crisis and damage amplifier

Mitochondria are central organelles where metabolic disturbances converge and amplify downstream damage signals. Lipotoxicity, hyperglycemia, and AGEs directly damage mitochondria, manifested by reduced membrane potential, impaired electron transport chain complex activity, decreased oxidative phosphorylation coupling efficiency, and reduced ATP synthesis (23, 24). Through the “metabolic memory” mechanism, the epigenetic modifications caused by high blood sugar persist even after blood sugar levels return to normal, leading to persistent mitochondrial dysfunction. In contrast, the “metabolic-structural coupling” phenomenon may extend to the mitochondrial level: metabolic molecules can physically interact with mitochondrial structural proteins, directly altering the dynamic characteristics of the mitochondria, going beyond the traditional signaling pathways. Mitochondrial dynamics are disrupted, with decreased fusion proteins (e.g. Mfn1/2) and increased fission protein Drp1, leading to excessive mitochondrial fragmentation (25–28).

Beyond mitochondria-derived ROS due to metabolic overload, hyperglycemia also activates the NADPH oxidase (NOX) family in the myocardium, contributing significantly to non-mitochondrial ROS production (29–31) Meanwhile, endogenous antioxidant defense systems are often downregulated or exhausted in diabetes. Mitophagy, the key mechanism for clearing damaged mitochondria (e.g. the PINK1/Parkin pathway), is frequently dysfunctional in DCM, leading to accumulation of defective mitochondria and further ROS generation—a self-perpetuating vicious cycle (32–36) Apart from energy failure, mitochondrial dysfunction may also contribute to “metabolic-structural coupling” by releasing metabolites that physically interact with myofilament proteins, thereby directly impairing the contractile ability.

### 2.3 Chronic low-grade inflammation and immune activation

Metabolic and oxidative stress jointly initiate a persistent, low-grade inflammatory state in the heart, serving as a critical bridge linking metabolic abnormalities to structural remodeling. Damage-associated molecular patterns (DAMPs) such as ROS and AGEs activate NF-κB, and the NLRP3 inflammasome, driving pro-inflammatory cytokine production (37–44).

Concomitantly, circulating monocytes are recruited to the diabetic heart and polarize toward a pro-inflammatory M1 phenotype, while reparative M2 macrophages are relatively reduced (45–49) These activated immune cells secrete large amounts of cytokines and growth factors (e.g. TGF-β), directly stimulating resident cardiac fibroblasts to transdifferentiate into matrix-producing myofibroblasts (50–53) Inflammatory signals themselves can worsen local metabolic disturbances; for example, TNF-α exacerbates myocardial insulin resistance, creating a positive feedback loop of “metabolic disturbance–inflammation–worsened metabolism”. (54, 55), This inflammatory network extends beyond the heart through interactions between organs. The adipose tissue releases adipokines that can directly regulate the inflammatory and fibrotic processes of the heart; while the liver produces hepatic factors that may exacerbate insulin resistance in the myocardium. Therefore, DCM is part of the systemic inflammatory network that connects the heart, fat, and liver.

### 2.4 Cardiomyocyte fate alterations: death, senescence, and dysfunction

Under persistent stress, cardiomyocytes undergo various fate changes. Programmed cell death in DCM includes apoptosis, necroptosis, pyroptosis, and ferroptosis—each with distinct molecular machinery and inflammatory consequences (56–63).

**Figure 1:**
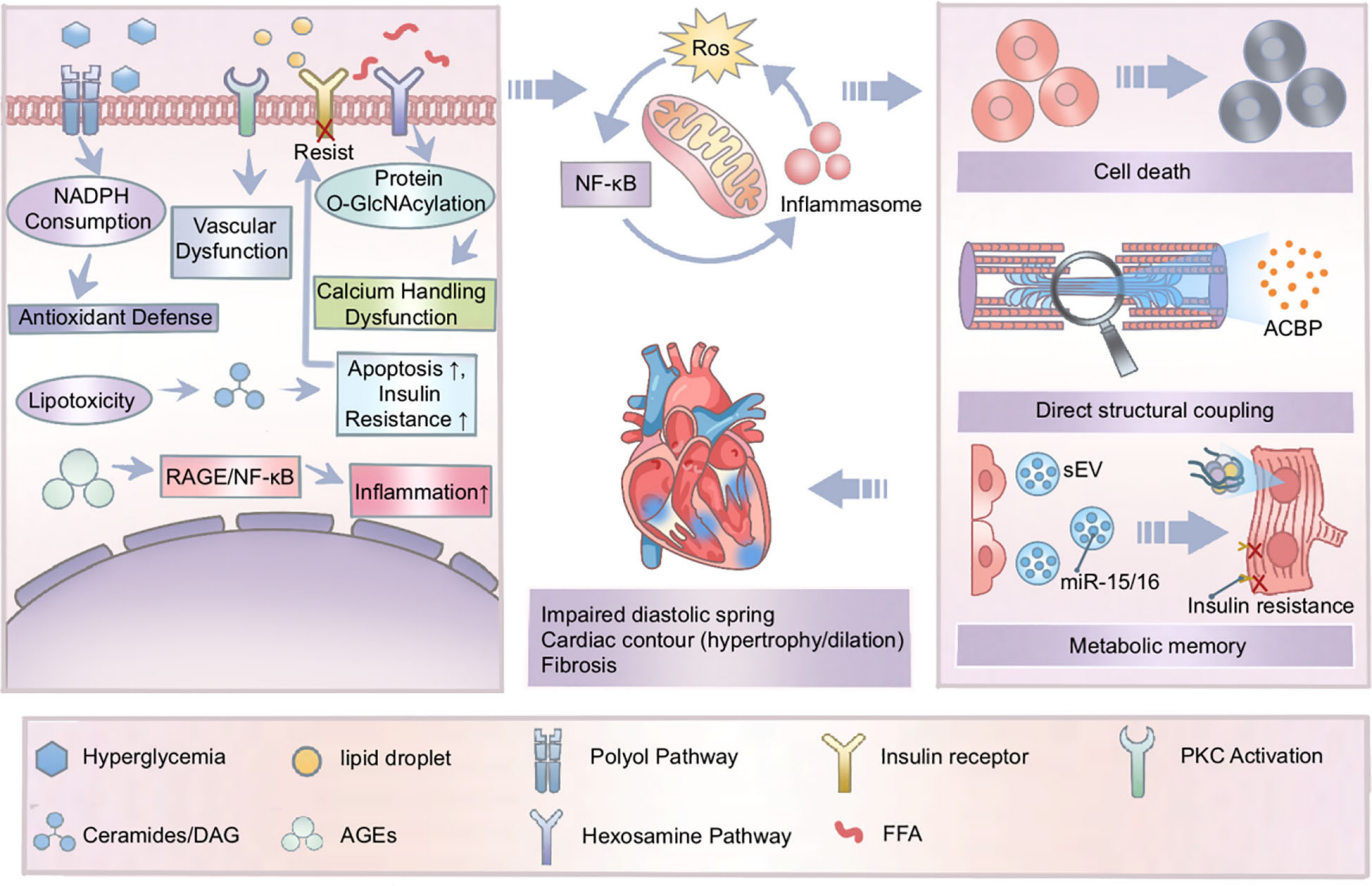
The interconnected pathophysiological network of diabetic cardiomyopathy (DCM). This schematic delineates a non-linear network where metabolic disturbances (glucolipotoxicity and AGEs) and cellular stressors (mitochondrial dysfunction, inflammation, and impaired calcium handling) form mutually reinforcing loops. These interactions jointly dictate cellular fates (senescence and cell death programs), which are sustained by “metabolic memory” to drive progressive structural remodeling. The integration of these unidirectional pathways creates a self-amplifying cycle that maintains chronic myocardial injury beyond a simple linear cascade.

Cellular senescence is also involved: metabolic stress accelerates the entry of cardiomyocytes and cardiac stem cells into a senescent state, characterized by the senescence-associated secretory phenotype (SASP) (64–66) SASP further deteriorates the local microenvironment, promoting fibrosis and dysfunction (67, 68). At the functional level, these stresses directly impair sarcoplasmic reticulum function, reducing activity of SERCA2a and disrupting calcium handling—the most direct cellular physiological basis for contractile dysfunction (69, 70). This complex interaction can be understood as a multi-level network (**Figure 1**). The progression of the disease begins with metabolic bypasses driven by glycolipid toxicity, such as the polyol pathway and the formation of AGEs, and then is exacerbated through a bidirectional interaction between mitochondrial oxidative stress and inflammation. Eventually, these interrelated disorders ultimately lead to irreversible structural remodeling and the occurrence of multiple cell death programs, including ferroptosis and senescence, which are mediated by key molecular coupling mechanisms. Unlike a linear cascade, this network has reinforcing relationships between all levels, without a single “starting point” or “endpoint”. The schematic emphasizes that DCM pathogenesis represents not linear toxicity from individual pathways, but rather a dynamic, self-amplifying network of molecular and cellular events wherein metabolic disturbances, structural disruption, inflammatory signaling, and epigenetic memory continuously reinforce one another to drive disease progression.

### 2.5 Cardiac autonomic neuropathy

Diabetes frequently leads to cardiac autonomic imbalance, characterized by increased sympathetic tone and reduced parasympathetic activity (71). Long-term sympathetic overactivation via β-adrenergic receptors increases myocardial oxygen consumption, promotes pathological hypertrophy and fibrosis, and predisposes to arrhythmias—an important component of the overall DCM pathophysiological network (72–77).

## 3 Networked interactions and emerging integrative concepts

### 3.1 Cross-talk and feedback loops among core mechanisms

The pathogenesis of DCM is a dynamic system with extensive cross-talk. Oxidative stress and inflammation amplify each other: ROS activate NF-κB and the NLRP3 inflammasome; conversely, activated inflammatory cells produce large amounts of ROS (57, 78–81) Metabolism and inflammation engage in vicious cross-talk: inflammatory cytokines induce local insulin resistance, while inflammation-driven fibrosis alters myocardial stiffness and perfusion, exacerbating energy crisis (82–84) Cell death and inflammation promote each other: necroptosis releases DAMPs, activating immune responses, while a persistent inflammatory milieu makes surviving cardiomyocytes more prone to cell death (57). Mitochondria serve as a central hub, releasing molecules (e.g. mitochondrial DNA) that act as DAMPs, thus connecting metabolic disturbances, oxidative stress, and inflammation (85, 86). Metabolic-structural coupling and metabolic memory are also interrelated, because directly disrupting myofilament proteins may lead to epigenetic changes, while continuous epigenetic modifications may enable the expression of pathogenic metabolic molecules to be maintained. A comprehensive visualization of these networked interactions and emerging concepts is provided in **Figure 1**.

### 3.2 “Metabolic–structural coupling”: from signal interference to direct physical disruption

The emerging “metabolic–structural coupling” hypothesis proposes a direct injury model. A recent study found that in diabetic myocardium, the lipid metabolism molecule acyl-CoA binding protein (ACBP) is upregulated and aberrantly binds to the core sarcomeric structural protein, cardiac myosin-binding protein C (MyBPC3). This abnormal binding physically interferes with MyBPC3’s function, directly compromising cross-bridge cycling efficiency (87). This discovery provides a new dimension for understanding how metabolic disturbances directly lead to decreased myocardial mechanical performance. This concept suggests that physical interactions between metabolites and structural proteins could serve as novel biomarkers for early DCM diagnosis, beyond soluble signaling molecules. Moreover, targeting ACBP or its binding interface with MyBPC3 might offer a therapeutic strategy to directly restore myocardial contractility, independent of traditional metabolic or inflammatory pathways. In addition to the acute insulin resistance plasma protein (ACBP), intermediate products of glycolysis such as methylglyoxal and lactic acid can directly modify myofilament proteins through glycosylation and lactoylation. The lactoylation of histones driven by lactic acid provides a direct link to the metabolic memory mechanism. In this mechanism, these epigenetic marks persist even after blood sugar returns to normal and encode long-term functional disorders.

### 3.3 “Metabolic memory”: the persistent imprint of injury and epigenetic basis

The “metabolic memory” phenomenon, where tissue injury progresses even after normalization of hyperglycemia, is a major clinical challenge. A key pathway has been revealed: hyperglycemia induces sustained release of small extracellular vesicles (sEVs) enriched with the miR-15–16 cluster from endothelial cells. These vesicles are taken up by cardiomyocytes, leading to long-term activation of CaMKIIδ and increased O-GlcNAc modification, persistently driving dysfunction (6). Intensive glucose lowering in late-stage DCM may offer diminishing returns because the memory is already “encoded” epigenetically. This positions early glycemic control and interventions that reverse epigenetic marks as priority research avenues. Beyond the heart, similar interactions mediated by extracellular vesicles are likely to occur between the heart of diabetic patients and distant organs (liver, adipose tissue, skeletal muscle). These interactions will spread metabolic memory throughout the body, thereby explaining why dilated cardiomyopathy often coexists with other diabetic complications. This explains the limited benefit of late intensive glucose-lowering and underscores the critical importance of early intervention. From a clinical perspective, metabolic memory highlights the need for risk stratification based on epigenetic markers. Circulating sEVs carrying specific miRNA signatures could serve as non-invasive biomarkers to identify patients at high risk for progressive DCM despite glycemic control. Therapeutically, interventions that block sEV generation or uptake, or reverse detrimental epigenetic modifications, may break the memory loop and halt disease progression. The ATAC-seq study in the model of dilated cardiomyopathy revealed the dynamic changes in chromatin accessibility, which were associated with the changes in gene expression in metabolic and contractile pathways. This provided a direct molecular basis for how metabolic memory is “encoded” at the chromatin level and offered the possibility of potential epigenetic targets for therapeutic reversal. Therefore, metabolic memory has the ability to integrate the states of the entire system at different time points. This characteristic explains why metabolic disorders that occur early, even after blood sugar levels return to normal, still persist and cause mitochondrial dysfunction and inflammatory responses. From a clinical perspective, this mechanism of metabolic memory prompts us to redefine dilated cardiomyopathy as a disease with “time-dependent” characteristics. In such diseases, early intervention measures can achieve particularly significant therapeutic effects.

## 4 Clinical heterogeneity of DCM: rethinking mechanisms and therapeutic implications

DCM is not a homogeneous disease. In type 1 diabetes (T1DM)-related DCM, mechanisms may be more focused on “glucotoxicity”. (88) Therefore, early strict glycemic control may be more decisive for prevention (89). In contrast, type 2 diabetes (T2DM)-related DCM is more prominently characterized by “insulin resistance” and “lipotoxicity”. (90–92).

Age also influences DCM. Early-onset diabetes exposes patients to metabolic abnormalities for longer, making “metabolic memory” effects more significant (93–95) In late-onset diabetes, DCM interacts with age-related cardiac changes, making the disease course more complex (96–99) Comorbidities like obesity and chronic kidney disease (CKD) are significant aggravating factors, forming a complex cardiorenal–metabolic syndrome (100–108).

## 5 Emerging therapeutic strategies

First, it must be recognized that lifestyle intervention measures such as calorie restriction, aerobic exercise, resistance training, and weight management remain the key foundation for the prevention and management of dilated cardiomyopathy. These interventions can directly enhance myocardial sensitivity to insulin, reduce lipotoxicity, and alleviate oxidative stress and inflammation. Weight loss can improve diastolic function in patients with obesity and diabetes. Based on the following treatment framework, which is the adjustment of lifestyle, drug therapy and experimental strategies are superimposed. Given the clinical heterogeneity discussed above, therapeutic approaches must be tailored to individual patient profiles. For example, obese T2DM patients may particularly benefit from therapies targeting lipid droplet dynamics and farnesoid X receptor (FXR) activation, while elderly patients with cellular senescence might be prime candidates for senolytics. Below we review the key emerging strategies.

### 5.1 Pleiotropic network effects of SGLT2 inhibitors

SGLT2 inhibitors (SGLT2i) exhibit multilayered network regulation. Their direct effects include inhibiting renal SGLT2 and myocardial sodium-hydrogen exchanger (NHE), improving energy metabolism (109). At an intermediate level, they activate autophagy, restore mitophagy, and upregulate NRF2, thereby inhibiting ferroptosis (32, 33). These actions lead to downstream outcomes of reduced inflammation and fibrosis. Clinical trials like EMPA-REG OUTCOME, DAPA-HF, and others consistently demonstrate their cardiovascular benefits (**Table 1**). SGLT2i represent a prototypic network-based therapy that simultaneously targets multiple core DCM pathways. However, most of the experimental data come from patients who already have heart failure, rather than from patients with early DCM. This creates a critical gap in primary prevention. The challenges in the translational aspect mainly lie in the lack of comprehensive preclinical models that fully represent the heterogeneity of human DCM, as well as the uncertainty regarding the optimal dose. Emerging evidence suggests SGLT2i may reverse certain epigenetic marks associated with metabolic memory, though direct effects on metabolic-structural coupling remain unexplored. Additionally, there is an urgent need to conduct randomized controlled trials and the determination of mechanistic biomarkers.

**Table 1:** Key clinical trial evidence for SGLT2i and GLP-1RA in diabetic populations.

Therapy class	Key trials	Main CV outcomes (DCM-relevant)
SGLT2 inhibitors	EMPA-REG OUTCOME (empagliflozin), DAPA-HF (dapagliflozin)	HF hospitalization ↓, CV death ↓, benefit independent of glucose lowering
GLP-1 receptor agonists	SOUL Randomized Trial	MACE ↓, atherosclerotic events ↓, modest effect on HF hospitalization

### 5.2 Cardiovascular protective effects of GLP-1 receptor agonists

GLP-1 receptor agonists (GLP-1RAs) reduce inflammation and oxidative stress, inhibit apoptosis, and improve endothelial function (110, 111). Clinical data show they reduce major adverse cardiovascular events (MACE), particularly atherosclerotic events, though their effect on heart failure hospitalization is less pronounced than SGLT2i (**Table 1**; 112–117) This differential efficacy underscores the need to match therapy to patient phenotype: GLP-1RAs may be preferred in DCM patients with predominant macrovascular disease, whereas SGLT2i might be more suitable for those with heart failure risk. At present, there are significant clinical trial gaps in the relevant research field, namely, no specific clinical trials have been conducted that focus on the target endpoints of DCM (without considering major vascular events) as evaluation indicators. Moreover, the translational application potential of GLP-1RAs in the treatment of DCM is limited by two factors. One is the issue of gastrointestinal tolerance in patients, and the other is the lack of preclinical models that can precisely reproduce the heterogeneity of human DCM. In particular, it is important to note that the DCM patients with normal weight constitute a large subpopulation, and currently there is a lack of clear clinical evidence to support the relevant treatment strategies.

### 5.3 Emerging therapies targeting ferroptosis

Irisin, a myokine, has been shown in preclinical studies to inhibit ferroptosis by activating the System Xc-/GSH/GPX4 axis (118, 119). Other muscle cell factors and liver factors also influence the progression of dilated cardiomyopathy. In this systemic network, skeletal muscles and the liver communicate with the heart through endocrine mediators. Alpha-lipoic acid (ALA) demonstrated significant efficacy in a small randomized controlled trial in patients with T2DM and ischemic cardiomyopathy, reducing inflammatory and fibrotic markers and improving cardiac function (120). Given that ferroptosis may play a more prominent role in advanced DCM, these agents could be particularly valuable for patients with established heart failure. However, the ALA trial was of a relatively small scale (with a sample size of only 67 cases), and it was conducted in patients with ischemic cardiomyopathy rather than purely dilated cardiomyopathy patients. The key gaps in translational research include the lack of specific ferroptosis biomarkers for patient screening, as well as the unclear long-term safety of chronic GPX4 pathway regulation. Whether ferroptosis inhibitors affect metabolic-structural coupling (e.g. by reducing ACBP expression or preventing its binding to MyBPC3) or reverse epigenetic memory marks remains unknown and warrants future investigation.

### 5.4 Senolytics: clearing senescent cells

Senolytics like dasatinib and quercetin (D+Q) have shown promise in animal models of DCM, clearing senescent cells and alleviating SASP-related inflammation (121, 122). Direct clinical evidence in DCM is insufficient, but trials in diabetic kidney disease support the safety and feasibility of this approach (123). In late-onset diabetes, where cellular senescence is accelerated, senolytics could mitigate age-related cardiac deterioration and improve regenerative capacity. Despite this, direct clinical evidence in DCM is absent, no trial has reported DCM-specific outcomes. Major limitations include off-target effects on non-senescent cells, unknown dosing regimens for cardiac versus renal indications, and the lack of validated senescence biomarkers to guide therapy initiation and monitoring. Senolytics could theoretically interrupt metabolic-structural coupling by eliminating senescent cells that secrete ACBP or other pathogenic metabolites, and may also reset epigenetic clocks, but direct experimental evidence is lacking.

### 5.5 Emerging targets: lipid droplet dynamics and FXR receptor

Targeting lipid droplet homeostasis is a new frontier (124, 125). Similarly, the farnesoid X receptor (FXR) is a key regulator of metabolism and inflammation. Obeticholic acid (OCA), an FXR agonist, has shown cardioprotective effects in preclinical models by activating the FXR/Nrf2 pathway (126–128). For obese T2DM patients with pronounced lipotoxicity, modulating lipid droplets and activating FXR might synergistically alleviate cardiac lipid overload and inflammation.

## 6 Conclusions and future directions

DCM pathogenesis has evolved into a complex dynamic network. The emerging concepts of “metabolic–structural coupling” and “metabolic memory” reveal underlying reasons for the limitations of previous therapies. Targeting ferroptosis, cellular senescence, and metabolic regulators like FXR are promising new directions.

However, these strategies face challenges: most targets lack human validation, clinical trial evidence is often from small samples, and the clinical heterogeneity of DCM demands better biomarkers for precise stratification. Within this framework, metabolic-structural coupling explains how metabolic stress directly impairs contraction, while metabolic memory accounts for disease persistence despite glucose control—together bridging molecular pathways to clinical phenotypes. Future research must apply multi-omics technologies to human samples and develop more representative disease models to accelerate preclinical validation. The ultimate goal is a paradigm shift from “one disease, one treatment” to “one disease, multiple subtypes, personalized therapy”.
